# Adsorptive removal of the cationic dye malachite green from wastewater with an anionic hydrogel

**DOI:** 10.1039/d6ra05581h

**Published:** 2026-08-24

**Authors:** Sawera Safdar, Muhammad Ajmal, Muhammad Abid Zia, Kashif Ali Khan, Afzal Shah

**Affiliations:** a Department of Chemistry, Division of Science and Technology, University of Education Lahore Pakistan abid@ue.edu.pk; b Department of Chemical and Environmental Engineering, University of Nottingham Ningbo 315100 China; c Department of Chemistry, Quaid-i-Azam University Islamabad 45320 Pakistan afzals_qau@yahoo.com

## Abstract

Contamination of water with various hazardous chemicals is increasing due to the rapid growth of industries, which has devastating effects on all living organisms. Thus, the decontamination of water has become crucial. This work reports the removal of a toxic cationic dye, malachite green (MG), from water using a bulk anionic hydrogel synthesized specifically for this purpose. The hydrogel was fabricated through the free radical polymerization of 2-acrylamido-2-methylpropane sulfonic acid (AMPS) and acrylamide (AAm) employing *N*,*N*′-methylenebisacrylamide (MBA) as the crosslinking agent. FTIR spectroscopic analysis confirmed the formation of the p(AMPS-*co*-AAm) hydrogel. Structural characterization using SEM revealed a well-interconnected porous network, supporting efficient dye uptake. Swelling analysis demonstrated a maximum equilibrium swelling of 2009.49% and a water content of 95.35%, with water transport following a non-Fickian transport mechanism. Adsorptive removal of the MG dye was investigated under varying conditions of hydrogel dosage, initial dye concentration, contact duration, and solution pH. A maximum adsorption capacity of 930.31 mg g^−1^ was recorded at 400 ppm, while 99.5% removal of the dye was achieved at 180 ppm. The adsorption data were best fitted to the Langmuir isotherm, which confirmed monolayer adsorption, while kinetic analysis aligned with the pseudo-first-order (PFO) kinetic model, indicating a physisorption-governed rate mechanism. The adsorption process was found to be strongly pH dependent, with a maximum uptake of 302.33 mg g^−1^ and an efficiency of 98.99% achieved at pH 11, attributed to the enhanced electrostatic interactions between the deprotonated anionic hydrogel surface and the cationic MG molecules. The synthesized p(AMPS-*co*-AAm) hydrogel exhibited an ultrahigh adsorption capacity for malachite green, demonstrating its potential as an efficient adsorbent for wastewater treatment. Collectively, these results establish the bulk p(AMPS-*co*-AAm) hydrogel as a highly effective and scalable adsorbent for the removal of MG and other cationic contaminants from wastewater.

## Abstract 2

Contamination of water with various hazardous chemicals is increasing due to the rapid growth of industries, which has devastating effects on all living organisms.

## Graphical abstract

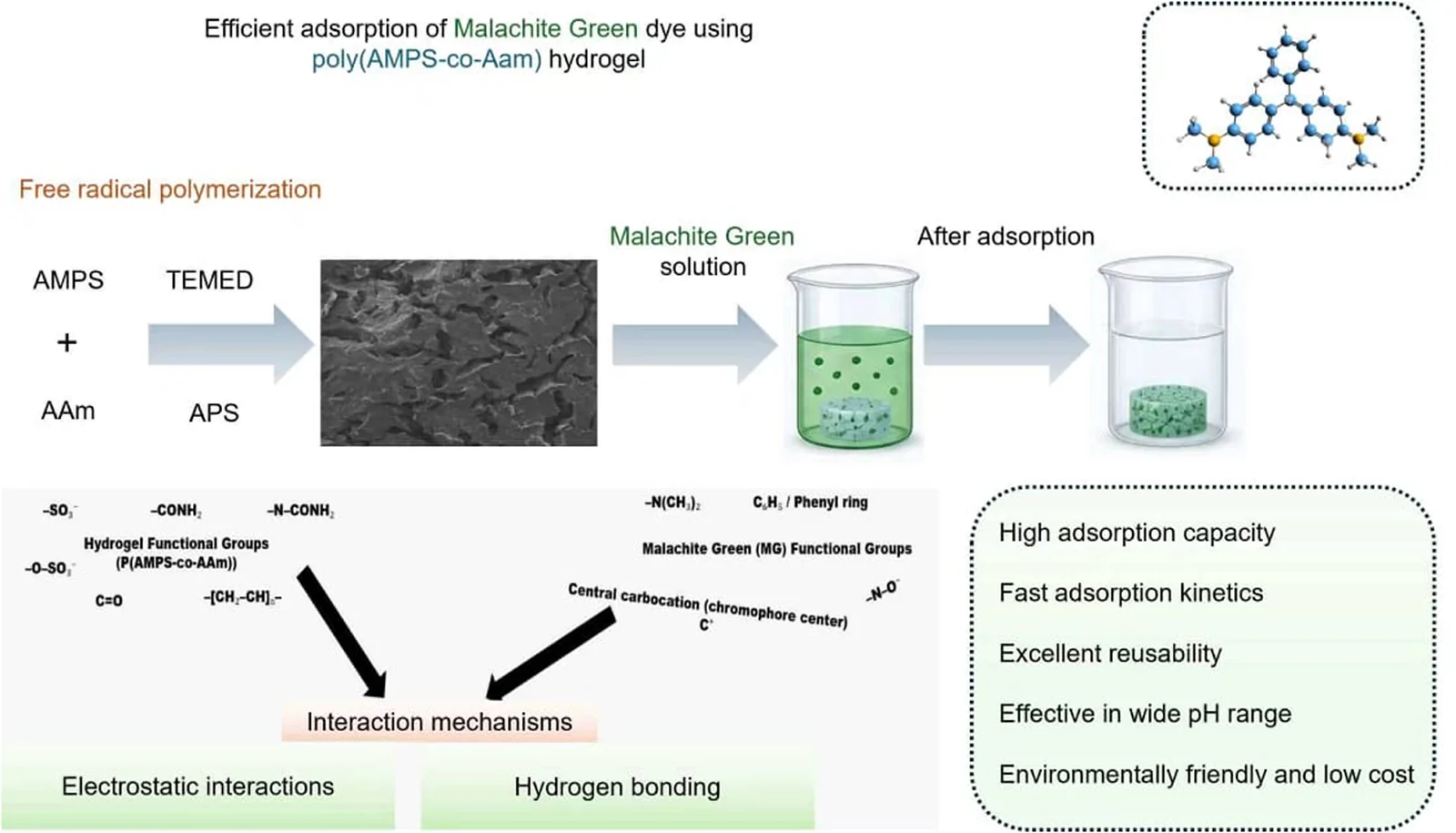

## 1. Introduction

Water is a vital resource for supporting life on earth, but its access in a pure state encounters considerable obstacles. Despite water encompassing 71% of the Earth's surface, merely 2.5% is deemed fresh water. Moreover, under 1% of this restricted supply is available to people *via* rivers, lakes, and groundwater.^1–3^ Rapid population growth, increasing urbanization, and accelerating industrial activity have placed unprecedented pressure on these fresh water resources, with synthetic dye-born effluents emerging as one of the most persistent and ecotoxicologically significant category of water pollutants.^4,5^ Around 1 million tons of synthetic dyes are produced worldwide every year and nearly 280 000 tons of these colorants are discharged in textile effluents.^6,7^ Synthetic dyes are considered refractory because they are organic compounds that can persist in water bodies for long durations and do not decompose quickly.^8^ Dye contamination has turned into an urgent worldwide environmental issue as even slight amounts can significantly change the ecological and visual properties of water, rendering the pollution easily observable.^9^ Beyond ecological damage, the food chain and human health consequences are profound. Notably, cationic dyes can cause skin irritation, mutagenicity, carcinogenicity, and teratogenicity, posing risks to aquatic ecosystems and human health.^10,11^ As a result, developing efficient and scalable methods for dye removal is critically necessary for scientific investigation and public health.

Malachite green (MG), a chemically *N*-methylated diaminotriphenylmethane, is a commonly used cationic dye in several sectors, including textiles, leather, paper, pharmaceuticals, and aquaculture.^12^ In solution, it appears as a mixture of a carbinol base and its colored cationic variant, referred to as chromatic malachite green, with the ratios differing based on the solution's pH. It is categorized as a Class II health hazard due to its harmful impact on essential organs, such as the liver, spleen, kidneys, and heart, along with its ability to induce irritations in the skin, eyes, and lungs.^13,14^ Furthermore, MG is not biodegradable, which enables it to persist in the environment, enter the food chain, and cause genotoxic, mutagenic, and carcinogenic effects in living beings.^15,16^ Despite numerous regulatory agencies prohibiting its application in aquaculture due to associated risks, MG remains in unlawful usage owing to its low cost and wide-ranging sterilizing effects. As a result, developing efficient and economical techniques for treating wastewater contaminated with malachite green remains a pressing and continuous challenge.

A range of methods, such as photocatalytic degradation, Fenton oxidation, chemical coagulation, flocculation, membrane filtration, ion exchange, and adsorption, have been utilized for dye removal.^17–22^ Nonetheless, numerous techniques suffer from considerable drawbacks, including elevated operational expenses, the production of secondary pollutants, and insufficient removal effectiveness. In comparison, adsorption is recognized as a very efficient decontamination method because of its affordability and quick kinetics and the absence of secondary pollution.^21,23^ The mass transfer process is essential to every physical pollutant removal technique, making adsorption particularly attractive due to its straightforwardness, versatility, effectiveness, and reusability. Its simple use and affordability have attracted increasing attention from researchers. As a result, adsorption emerges as an ideal option for removing dyes in both lab and industrial environments, with the selection of adsorbent material being vital to the efficiency of any adsorption-based removal system.

Arabic gum, guar gum, and polymer matrix-crosslinked chitosan hydrogels are some examples of biosorbents that are most commonly used.^24^ Their tunable chemistry, high internal surface area in the swollen state and capacity for electrostatic interactions, van der Waals interactions, and hydrogen bonding with dye molecules make them particularly useful for pollutant adsorption.^25,26^ Nevertheless, large-scale applications of these adsorbents are hindered by many intrinsic limitations. The natural gums have poor mechanical strength, which leads to structural disintegration or deformation during the adsorption process.^27^ The poor mechanical strength frequently results in the deterioration of their structure during adsorption–desorption cycles, which in turn makes it challenging to reuse these adsorbents.^28^ In addition, their chemical instability under strong acidic and basic conditions further decreases their applicability in adsorption processes.^29^ To overcome these limitations, recent research has increasingly focused on the development of chemically synthesized copolymer hydrogels with improved chemical resistance, enhanced structural integrity, improved mechanical stability and fast uptake of adsorbates.

Among various hydrogel systems, copolymers incorporating AMPS and AAm are particularly promising.^30^ AMPS exhibits a strong anionic character over a wide pH range, while AAm imparts excellent hydrophilicity, biocompatibility and specific dye binding capacity through its amide functional group. The anionic character of AMPS containing hydrogels arising from sulfonate groups creates a strong electrostatic driving force for the uptake of cationic pollutants such as MG.

Previous work has explored AMPS- and AAm-based systems for cationic dye removal, though exclusively within composite- or biopolymer-grafted architectures. Yu *et al.* prepared a chitosan-grafted CS/P(AMPS-*co*-AM) hydrogel *via* glow-discharge-electrolysis plasma-initiated copolymerization and demonstrated impressive adsorption capacities of 1538.5 mg g^−1^ for MB and 917.4 mg g^−1^ for MG under optimal conditions (pH 5.8, 90 min), with adsorption data best described by the pseudo-second-order kinetics and Langmuir isotherm.^31^ While the performance of this system is noteworthy, the reliance on a chitosan backbone and a specialized plasma initiation technique introduces significant synthetic complexity, limits reproducibility, and constrains scalability for practical applications. Similarly, Kumawat *et al.* synthesized a guar/locust bean gum-grafted poly(acrylamide) hydrogel (GG/LBG-*g*-poly(AAm)) *via* free radical polymerization for the MG removal, achieving a removal efficiency of 98% under optimized conditions; however, the maximum adsorption capacity was limited to 52.96 mg g^−1^, considerably below the performance benchmark set for purely synthetic systems.^32^ While natural polymer backbones offer biocompatibility advantages, they introduce batch-to-batch variability in gum composition and complicate mechanistic interpretation of the adsorption process.

The present study addresses, for the first time, the synthesis and systematic evaluation of a bulk p(AMPS-*co*-AAm) hydrogel as an adsorbent for the removal of MG from aqueous solutions. The combination of 2-acrylamido-2-methylpropane sulfonic acid (AMPS) and acrylamide (AAm) is selected because the highly ionized sulfonate groups (–SO^3−^) in AMPS provide strong, permanent electrostatic attraction toward cationic dyes like malachite green across a wide pH range. Concurrently, the AAm segments enhance the hydrogel's mechanical integrity, hydrophilic swelling capacity, and complementary hydrogen-bonding networks, resulting in superior synergistic adsorption compared to conventional single-monomer hydrogels. The hydrogel was synthesized *via* free radical polymerization and characterized using SEM and swelling analysis. The adsorption performance was evaluated under varying conditions of adsorbent dosage, initial dye concentration, pH of the solution and contact time. Equilibrium data were analyzed using the Langmuir, Freundlich and Temkin isotherm models, while the kinetics was evaluated using the PFO and PSO models. The hydrogel achieved a maximum adsorption capacity of 930.31 mg g^−1^ with monolayer adsorption and PFO kinetics. The results establish a bulk p(AMPS-*co*-AAm) hydrogel as a high-capacity, easily synthesized adsorbent with strong potential for scalable cationic dye remediation.

## 2. Experimental

### 2.1. Materials

The details of various chemicals employed during the process are presented in Section S1 and Table S1. Section S2 provides the details of instruments used in this work, while Section S3 provides experimental details of swelling analysis.

### 2.2. Synthesis of the bulk hydrogel p(AMPS-*co*-AAm)

**Fig. 1:**
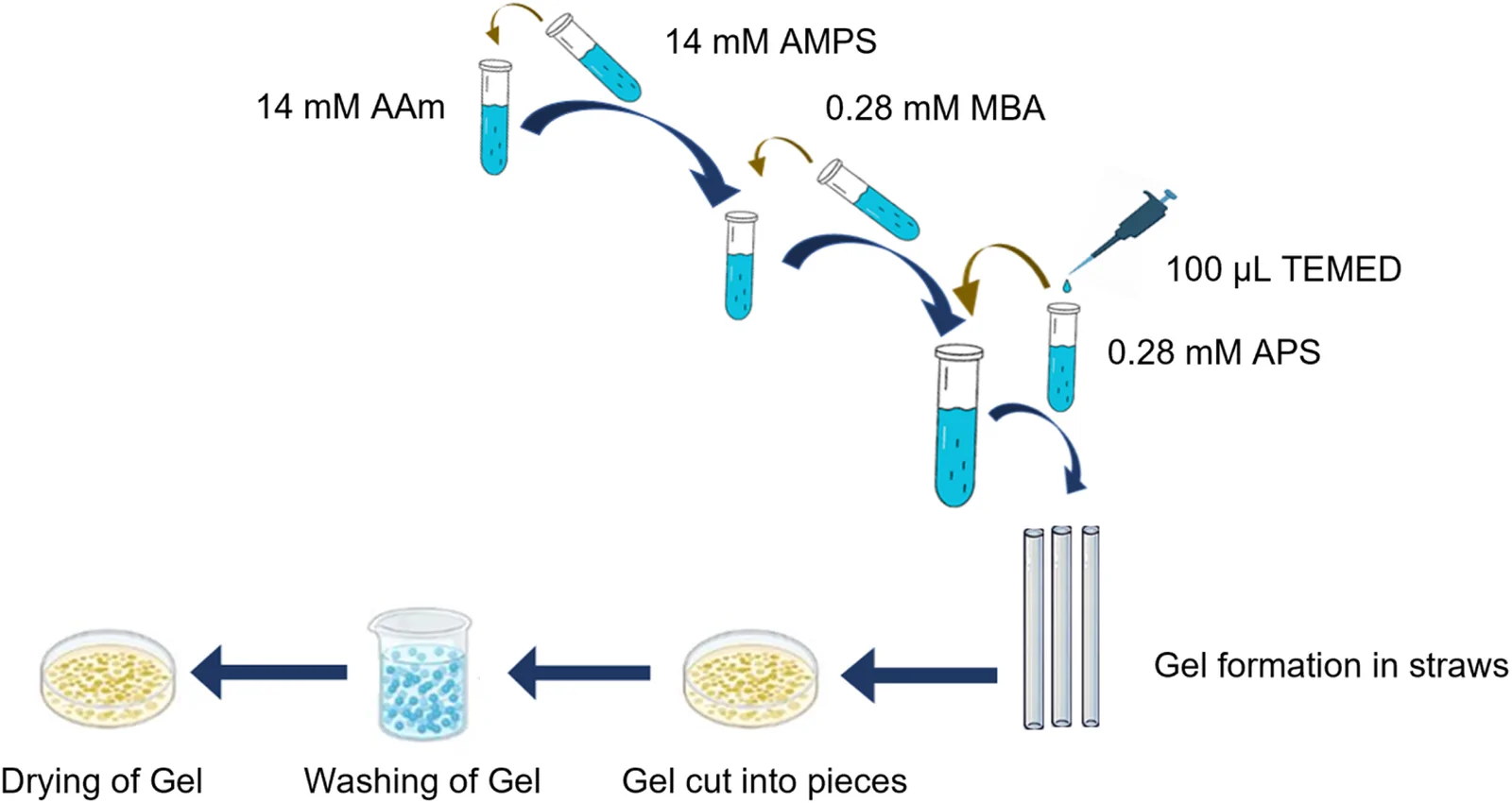
Schematic of the synthesis of the p(AMPS-*co*-AAm) polymer hydrogel.

The p(AMPS-*co*-AAm) hydrogel was prepared through free radical polymerization, with APS used as the radical initiator and TEMED as the accelerator, while MBA functioned as the cross-linking agent. In a typical reaction, AAm (14 mM) was first dissolved in a glass vial followed by the addition of AMPS (14 mM), which was allowed to dissolve completely to form a clear solution before the cross-linker MBA (0.28 mM) was introduced. In the second step, APS (0.28 mM) was prepared as a separate aqueous solution to which 100 µL TEMED was added using a micropipette and subsequently transferred into a monomer-cross-linker vial. To ensure homogeneity, the pre-gel solution was carefully mixed and poured into plastic straws. The addition of TEMED initiated polymerization by accelerating the decomposition of APS into sulfate radical anions, which, in turn, triggered chain-growth copolymerization of AAm and AMPS across the MBA crosslinks. Gelation was allowed to proceed at 25 °C for 24 hours to ensure full network formation. Once polymerization was confirmed, hydrogel rods were gently removed from the plastic straws and cut into little pieces. To get rid of any leftover monomers or unreacted compounds, the hydrogel was subjected to a thorough washing protocol for ten days using distilled water. To ensure complete cleaning, the water was changed twice a day. The purified hydrogel samples were then stored in sealed containers and used directly for swelling studies, adsorption measurements, and physicochemical characterization. Fig. 1 shows a step-by-step schematic of the synthesis of p(AMPS-*co*-AAm).

### 2.3. Adsorption experiments

Adsorption experiments were conducted using malachite green as a model cationic dye. The electrostatic attraction between the anionic sulfonate groups of p(AMPS-*co*-AAm) and the cationic MG molecule drives the adsorption of dyes onto the hydrogel network. The adsorption study was performed with laboratory-prepared dye solutions, and experiments were conducted under ambient conditions. Therefore, the absence of real wastewater experiments and thermodynamics of the adsorption process may be considered as a limitation of this work. However, the adsorption capacity under laboratory conditions was studied in detail. All adsorption experiments were conducted in triplicate, and the average values with standard deviations are reported.

#### 2.3.1. Calibration and stock solution preparation

A primary stock solution (910 ppm) was prepared by dissolving MG in distilled water. A working stock of 180 ppm was obtained by diluting the primary stock solution, and its maximum absorbance wavelength (*λ*_max_) was determined spectrophotometrically at *t*_0_. Calibration standards of 2, 4, 6, 8, and 10 ppm were prepared from the 180 ppm working stock by serial dilutions.

#### 2.3.2. Effect of hydrogel dose

Five 100 mL samples of the 180 ppm MG solution were transferred into separate 100 mL beakers (W1–W5). A different amount of p(AMPS-*co*-AAm) hydrogel (30, 50, 70, 90, and 110 mg) was added to each beaker and placed on a digital magnetic stirrer. At each sampling interval, 0.5 mL of solution was withdrawn and diluted with 4.5 mL of distilled water in a test tube prior to absorbance measurement. The samples were collected every 8 minutes during the first hour, then every 20 minutes in the second hour, and finally, every hour until equilibrium was established.

#### 2.3.3. Effect of the initial concentration

Working solutions of 120, 150, 180, 210, 240, 300, and 400 ppm were prepared from the 910 ppm stock solution. Each beaker contained 30 mg of hydrogel in 100 mL of the respective MG solution. The withdrawn aliquots (0.5 mL) were diluted with volumes of distilled water adjusted to account for the differing initial concentrations (6.5, 8.5, and 10 mL for 150, 180, and ≥210 ppm solutions, respectively) before absorbance measurement. Sampling followed the same time schedule as above. The concentration–time profiles were plotted to identify equilibrium for each initial concentration.

## 3. Results and discussion

### 3.1. Characterizations

#### 3.1.1. FTIR analysis

The hydrogel was synthesized by using AMPS and AAm as monomers and MBA as a cross linker. All these reagents contain C=C bonds in their structures. The synthesis was carried out by free radical polymerization, which utilized these C=C bonds as reactive sites for the growth of the polymer chain. As a result of polymerization, C=C bonds were converted into C–C bonds, which was indicated by the disappearance of the characteristic absorption band of C=C bonds in the FTIR spectrum of the p(AMPS-*co*-AAm) hydrogel, as shown in Fig. S1. The presence of the characteristic absorption bands of the sulphonic acid group (–S=O), amide (N–H bending and C=O stretching vibrations) and backbone (saturated alkyl C–H stretching) affirms the formation of the p(AMPS-*co*-AAm) hydrogel. In addition, the conversion of the watery reaction mixture into a semisolid inviscid material indicated the formation of the p(AMPS-*co*-AAm) hydrogel.

#### 3.1.2. Swelling behavior

**Fig. 2:**
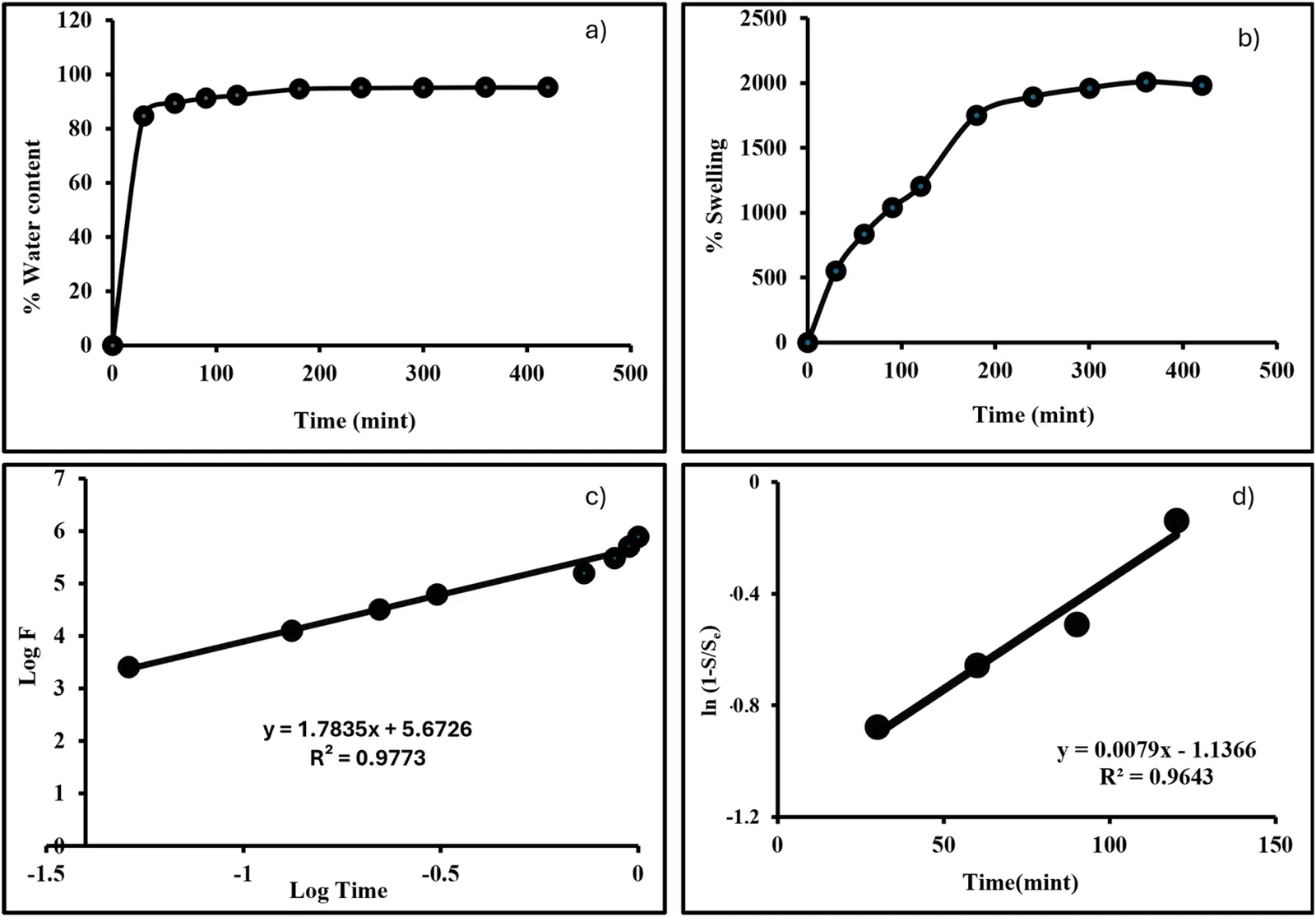
(a) Plot of % water content *vs.* time. (b) Swelling ratio represented by a plot of % swelling against time. (c) Plot of log *F vs.* log of time. (d) Plot of ln(1 − *S*/*S*_e_) *vs.* time of hydrogel synthesis.

The swelling behavior of the p(AMPS-*co*-AAm) hydrogel was evaluated by immersing dried specimens in distilled water at room temperature. Swollen samples were removed at regular intervals, surface dried to remove excess water and weighed until equilibrium was reached. The percentage swelling (%*S*) and water content (%WC) were calculated according to eqn (1) and (2),^33^ respectively, as follows:1
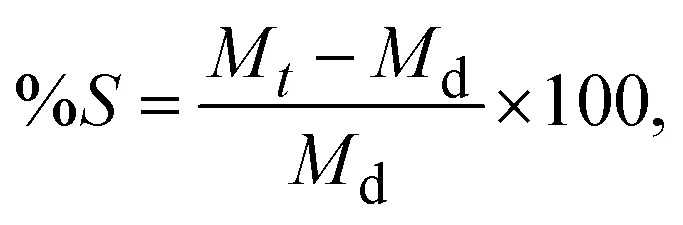
2
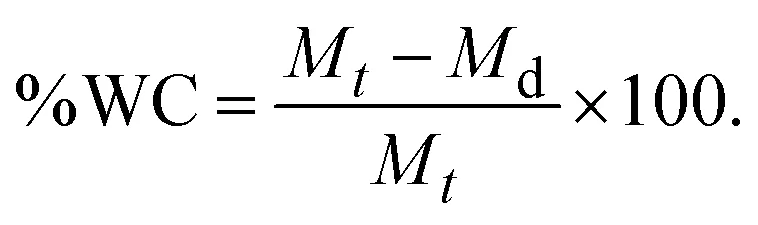
In these equations, *M*_*t*_ represents the mass of the hydrogel at time *t*, while *M*_d_ corresponds to dried hydrogel prior to immersion in water. The water content (%) was calculated, reaching 92.34% in only 2 hours, while a maximum of 95.25% was achieved in 6 hours period (Fig. 2a). It was observed that swelling increased rapidly for the first 30 minutes, reaching 553.16% and continued to increase steadily to a maximum of 2009.49% over the subsequent 6 hours, with equilibrium attained after approximately 31 hours (Fig. 2b). This high swelling capacity is achieved due to the hydrophilicity of the functional groups in the hydrogel and the optimal amount of the crosslinker. Upon swelling, the crosslinker prevents the breakdown of the polymeric network and its optimal low amount allows the maximum stretching of polymer chains, which, in turn, increases the adsorption capacity of the hydrogel.

To further explore the mechanism of water uptake, the classical power-law model (eqn (3)) was applied as follows:^34^3
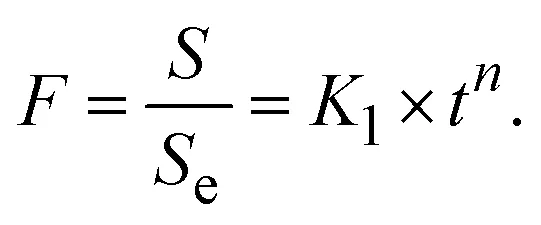
In this equation, *F* represents the swelling fraction, *S* corresponds to the swelling ratio at time *t* and *S*_e_ denotes the equilibrium swelling ratio. *K*_1_ is the network-dependent constant and *n* is the diffusional exponent, which can be determined from the slope of the log-linearized form of eqn (3), as shown in Fig. 2c. According to the literature, a value of *n* less than 0.5 indicates Fickian transport, a value within the range of 0.5–1 corresponds to anomalous transport, while a value greater than 1 represents non-Fickian transport mechanism. The calculated value of *n* suggests that the transport of the dye through the hydrogel network follows a non-Fickian transport mechanism, indicating that the swelling kinetics is governed primarily by the polymer chain rather than purely concentration gradient-driven diffusion.

The swelling rate was analyzed using the Voigt-based model (eqn (4)) as follows:^34^4
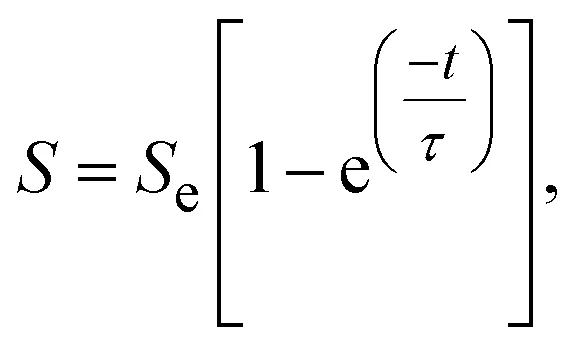
where *S* corresponds to the swelling ratio at time *t*, *S*_e_ represents the equilibrium swelling ratio at equilibrium, while *τ* is the swelling time constant (min), which reflects the resistance offered by the hydrogel to water diffusion. A lower value of *τ* corresponds to a faster swelling rate. The time constant was determined from the slope of the linearized logarithmic form of eqn (4) plotted against time, as shown in Fig. 2d, yielding *τ* = 126.5 min. This value characterizes the rate at which the hydrogel network accommodates water uptake, with elevated temperatures expected to reduce *τ* due to the increased chain mobility and accelerated water diffusion.

**Fig. 3:**
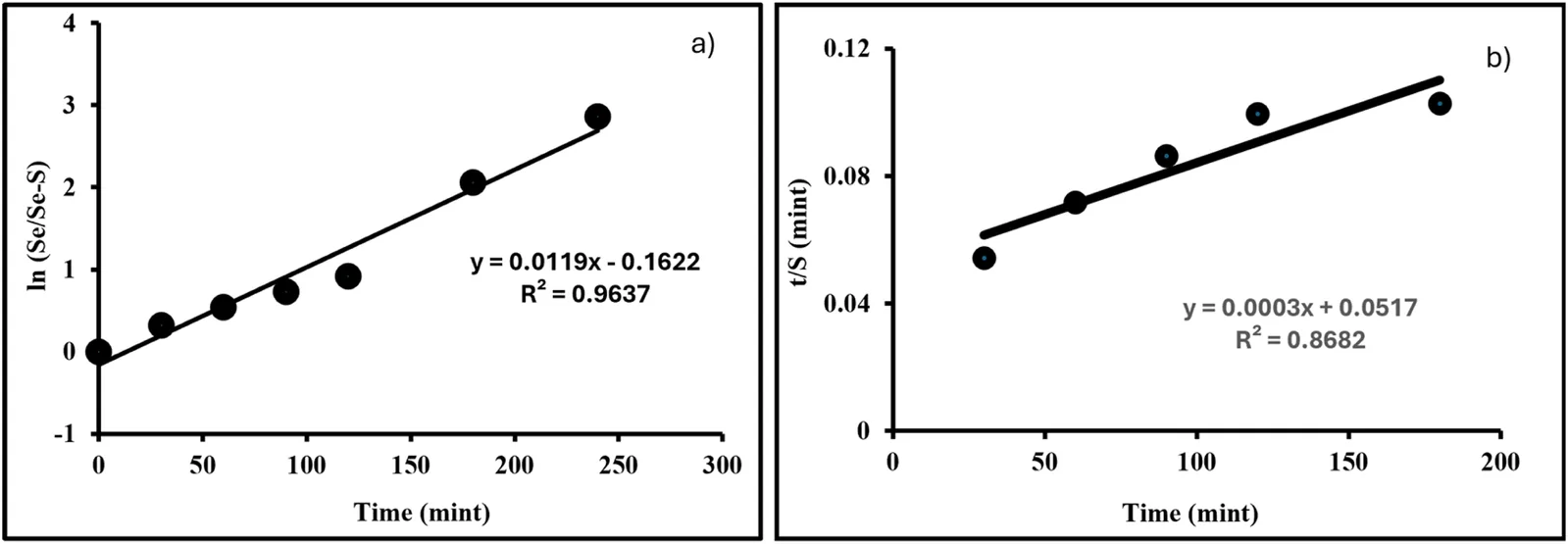
(a) First-order kinetic model plot of the adsorption of MG on the surface of the bulk p(AMPS-*co*-AAm) hydrogel. (b) Second order kinetic model plot of the adsorption of MG on the surface of the bulk p(AMPS-*co*-AAm) hydrogel.

Swelling kinetics were also assessed using first- and second-order kinetic models expressed as eqn (5) and (6), respectively, as follows:^35^5
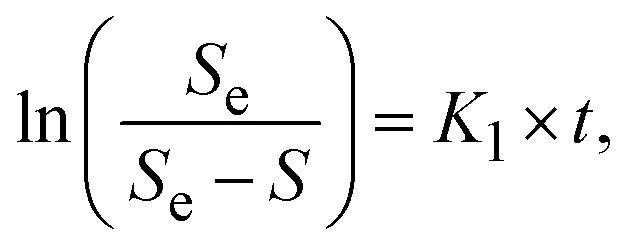
6
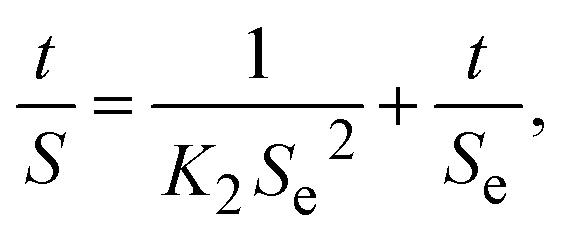
where *K*_1_ and *K*_2_ are the first- and second-order swelling rate constants, respectively. Linearity was assessed by plotting ln(*S*_e_/*S*_e_ − *S*) *versus t* for the first-order model and *t*/*S versus t* for the second-order model. The *R*^2^ values for the 1st- and 2nd-order kinetic models were calculated to be 0.964 and 0.868 (Fig. 3a and b), respectively, confirming the swelling kinetics of the p(AMPS-*co*-AAm) hydrogel as a first-order model, in which the swelling rate is proportional to the remaining swelling capacity of the network.

#### 3.1.3. SEM analysis

**Fig. 4:**
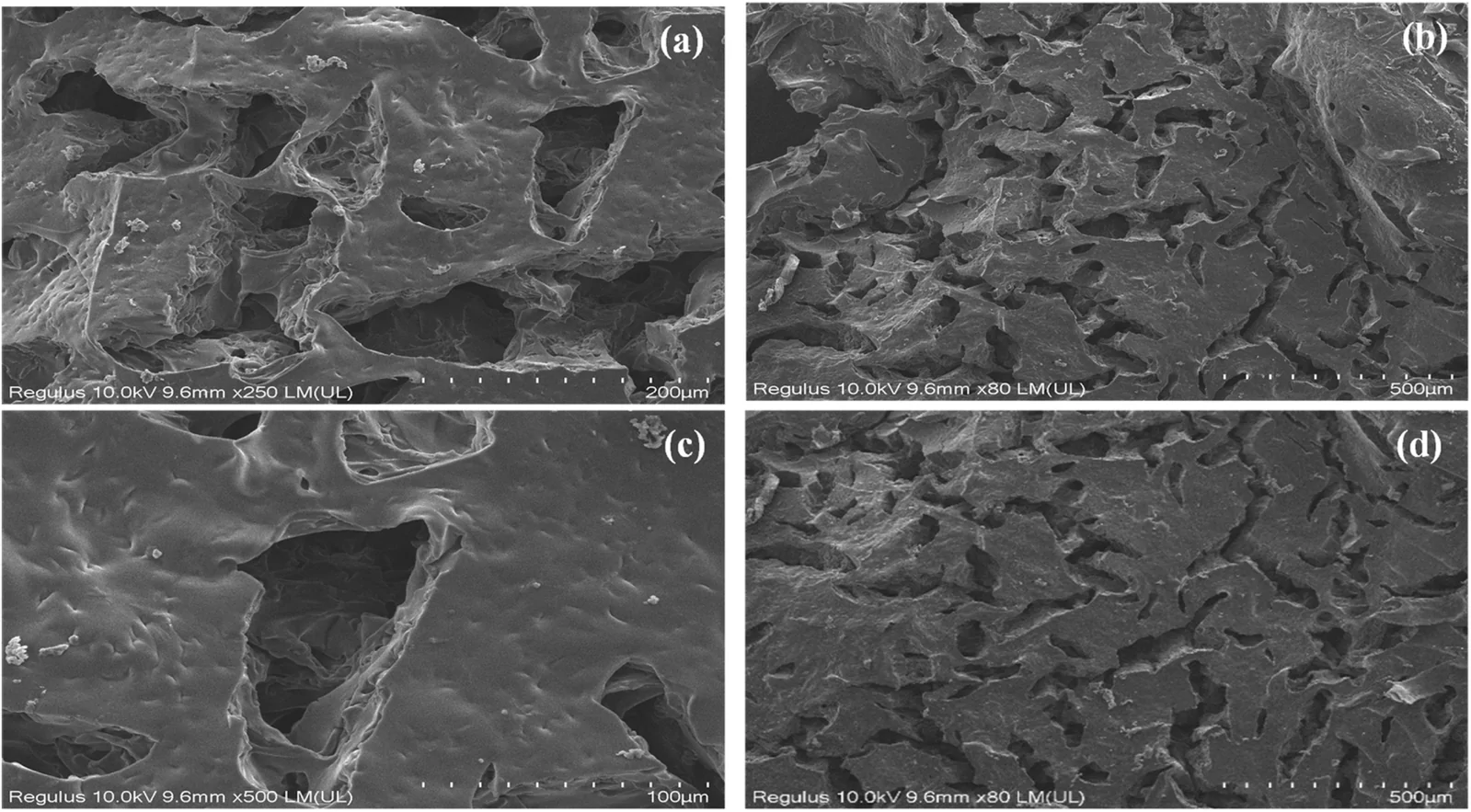
(a–d) SEM images of the p(AMPS-*co*-AAm) hydrogel under various magnifications.

The morphology of the synthesized p(AMPS-*co*-AAm) hydrogel was examined by scanning electron microscopy (SEM) at various magnifications, as shown in Fig. 4a–d. At a low magnification (×80, scale bar = 500 µm), the hydrogel exhibits a well-developed, interconnected porous architecture (Fig. 4a), which is characteristic of crosslinked hydrogel networks and is conducive to efficient water uptake and solute transport. Fine pores and membrane like texture of the network can easily be seen at a higher magnification (Fig. 4c), revealing a relatively uniform pore distribution. This feature is important particularly for maintaining mechanical integrity as well as controlled transport behavior. Fig. 4d represents the region of elevated cross-link density, which can be correlated with reduced water retention capacity. The presence of the sulphonic acid group from AMPS is expected to endow an anionic character to the pore surfaces, thereby enhancing electrostatic interactions with cationic species and contributing to the observed swelling and adsorption performance.

### 3.2. Adsorption study

The adsorption of MG from water was investigated using the synthesized p(AMPS-*co*-AAm) hydrogel. Because of the cationic nature of the malachite green dye and the presence of amide and sulfonic functional groups in the synthesized hydrogel, our prepared hydrogel was chosen as the adsorbent. As a result of the electrostatic forces of attraction, the malachite green dye may be attached onto the p(AMPS-*co*-AAm) hydrogel.

#### 3.2.1. Effect of hydrogel content

**Fig. 5:**
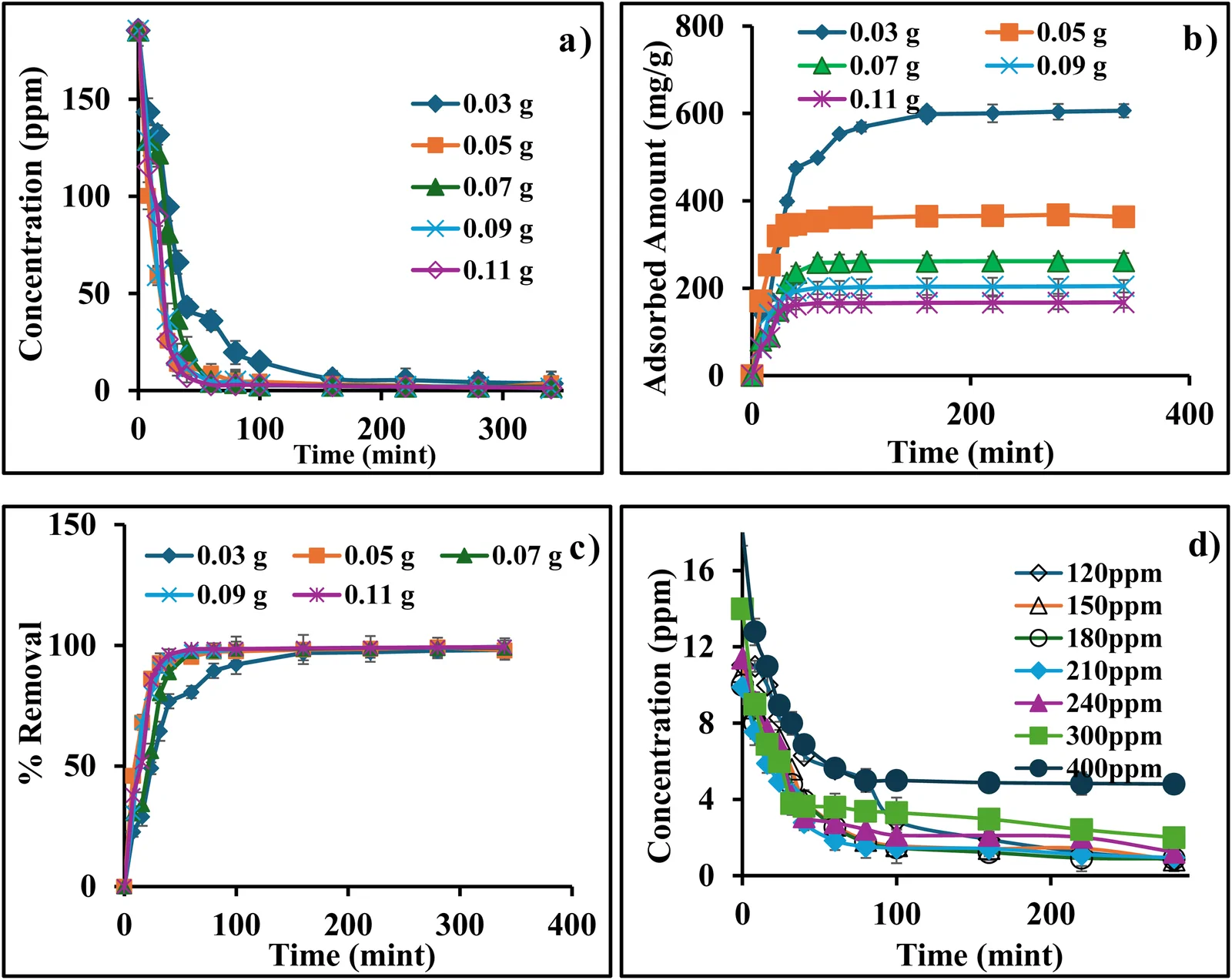
(a) Adsorption of MG onto the hydrogel surface at varying hydrogel concentrations. (b) Plot of the adsorption capacity (mg g^−1^) *vs.* time at various adsorbent dosages. (c) Plot of the removal efficiency (% removal) *vs.* time at various adsorbent dosages. (d) Adsorption of MG onto the adsorbent surface using different concentrations of the dye. Reaction conditions: 100 mL MG solution (180 ppm), 25 °C, 400 rpm, and hydrogel masses: 0.03, 0.05, 0.07, 0.09, and 0.11 g.

The impact of hydrogel content on MG removal was investigated by adding five different masses of p(AMPS-*co*-AAm) (0.03, 0.05, 0.07, 0.09, and 0.11 g) to 100 mL aliquots of a 180 ppm MG solution at 25 °C and 400 rpm. As represented in Fig. 5a, the dye concentration decreased progressively with time for all dosages, with a measurable decline observed within the first 24 minutes. Equilibrium was established at 340 minutes for all the samples. It was observed that the percentage of removal increased monotonically with the increasing hydrogel masses, which can be attributed to the increasing number of active sites upon increase in the adsorbent dosage (Fig. 5b). The maximum removal efficiency was calculated to be 99.30% at an adsorbent dose of 0.11 g, while the lowest removal efficiency was recorded at 0.03 g, confirming a strong dose-dependent response. Fig. 5c and d represent the plot of % removal and concentration as a function of time, respectively, both illustrating the rapid saturation of active sites.

#### 3.2.2. Impact of the initial dye concentration

**Fig. 6:**
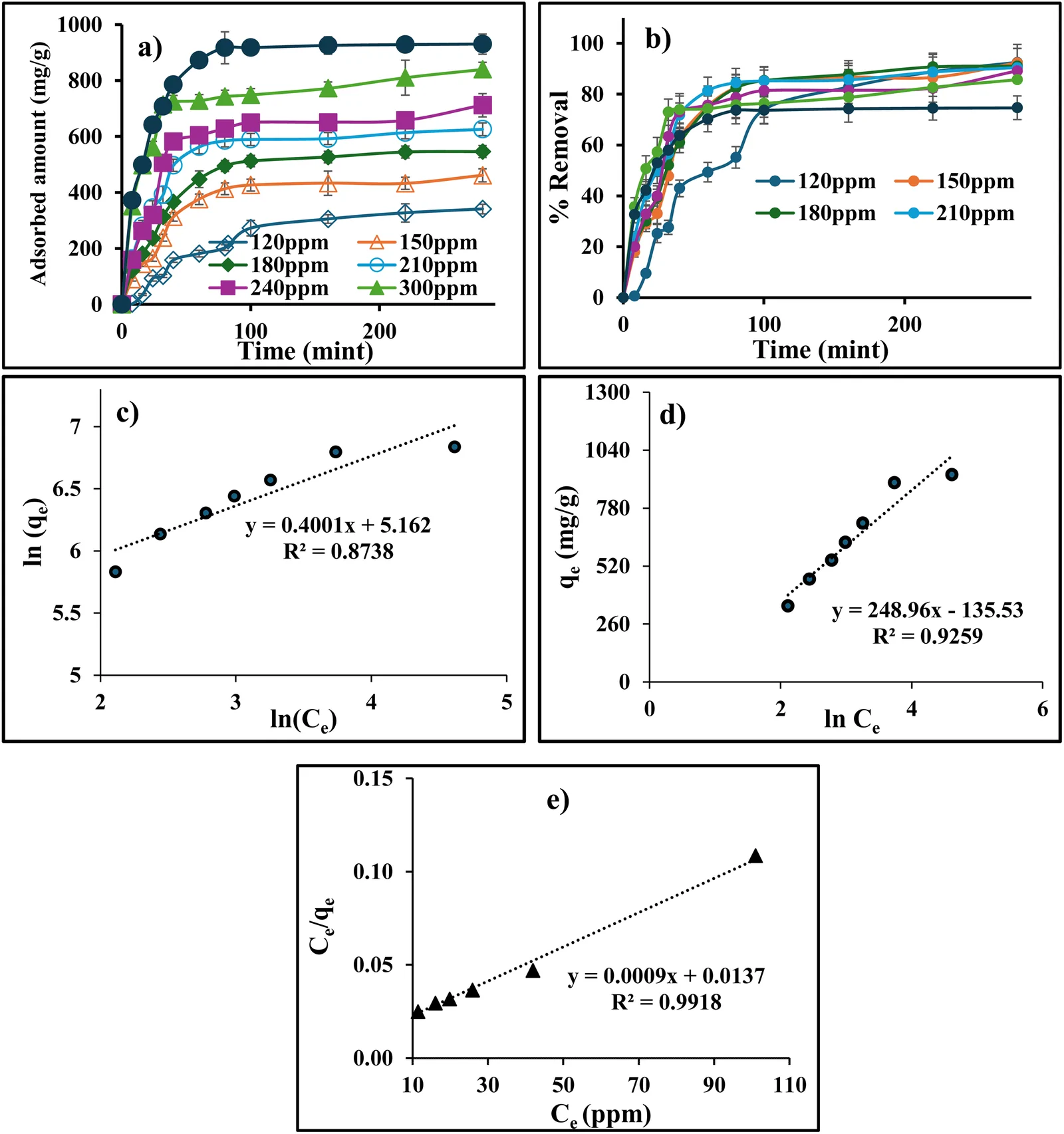
(a) Plot of the adsorption capacity as a function of time at varying initial dye concentrations. (b) Percentage of removal of the dye under the same conditions as those given in (a). (c) Freundlich adsorption isotherm. (d) Temkin adsorption isotherm. (e) Langmuir adsorption isotherm plotted as *C*_e_*/q*_e_*versus C*_e_. Reaction conditions: 0.03 g hydrogel, 100 mL MG solution, 25 °C, and 400 rpm.

To evaluate the impact of dye concentration on the adsorption capacity of the hydrogel, a fixed hydrogel dosage of 0.03 g was kept in contact with 100 mL solutions spanning concentrations of 120, 150, 180, 210, 240, 300, and 400 ppm at 25 °C and 400 rpm. The adsorption capacity (mg g^−1^) (onwards represented as *q*) increased with the increasing initial concentration, reflecting the greater concentration gradient that drives mass transfer from the solution to the hydrogel surface (Fig. 6a). Equilibrium was reached at 100 minutes for all concentrations. *q*_max_ of 930.31 mg g^−1^ was recorded at 400 ppm, demonstrating the exceptionally high uptake potential of the bulk hydrogel. Conversely, as represented in Fig. 6b, the percentage removal decreased with the increasing initial concentration, with near-complete removal (99.95%) achieved at 120, 150, and 180 ppm, where the available adsorption sites were sufficient to accommodate the dye load. At higher concentrations, the active sites became progressively saturated, limiting the removal efficiency. This inverse relationship between the initial concentration and the percentage removal is consistent with the site saturation behavior widely reported for hydrogel-based adsorbents.

#### 3.2.3. Isotherms of adsorption

The equilibrium adsorption data were analyzed using different isotherm models to determine the nature of MG adsorption onto the p(AMPS-*co*-AAm) hydrogel surface.

The Langmuir isotherm assumes monolayer adsorption on the equivalent surface with a finite number of energetically equivalent and non-interacting binding sites. The linear form of Langmuir isotherm (eqn (7)) is given as follows:^36^7
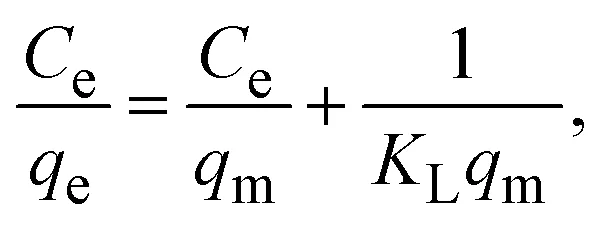
where *q*_m_ (mg g^−1^) is the theoretical maximum monolayer adsorption capacity, *C*_e_ (mg L^−1^) is the equilibrium dye concentration, *q*_e_ (mg g^−1^) is the equilibrium adsorption capacity, and *K*_L_ (L mg^−1^) is the Langmuir affinity constant. A plot of *C*_e_/*q*_e_*versus C*_e_ yielded a linear relationship with an *R*^2^ value of 0.9918 (Fig. 6e). The theoretical value of *q*_m_ calculated from this Langmuir model was also found to be close to the experimental value. A value of *R*^2^ close to 1 and resemblance among the theoretical and experimental values of *q*_m_ suggest the monolayer formation of MG on the hydrogel surface, and hence, a chemisorption mechanism.

The Freundlich isotherm accounts for multiple layered adsorption on heterogeneous surfaces and is expressed in linearized form (eqn (8)) as follows:^36^8
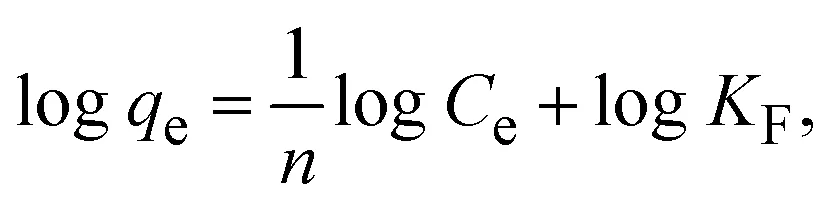
where *K*_F_ is the Freundlich adsorption capacity constant and 1/*n* is the heterogeneity factor. The values of 1/*n* < 1 indicate favorable adsorption, 1/*n* > 1 unfavorable adsorption, and 1/*n* = 1 irreversible adsorption. The parameters extracted from the ln *q*_e_*versus* ln *C*_e_ plot (Fig. 6c) are listed in Table S2. The low *R*^2^ value relative to the Langmuir model suggests that the Freundlich isotherm does not adequately describe the MG–hydrogel interaction, implying a predominantly homogeneous rather than heterogeneous adsorption surface.

The Temkin isotherm accounts for adsorbate–adsorbent interactions by assuming a uniform distribution of binding energies and a linear decrease in adsorption heat with surface coverage.^37^ Its mathematical form is given as eqn (9) as follows:

9 *q*_e_ = *B* ln *KT* + *B* ln *C*_e_,

where *B* (J mol^−1^) is a constant related to the heat of adsorption and *K*_T_ (L g^−1^) is the Temkin equilibrium binding constant. If the value of *B* is less than 1 kcal mol^−1^, it is indicative of physisorption, while its value between 1 and 20 kcal mol^−1^ suggests chemisorption. The Temkin plot of *q*_e_*versus* ln *C*_e_ is shown in Fig. 6d. Table S2 lists isotherm parameters for the adsorption of dye. Taken together, the isotherm analysis confirms that MG adsorption onto p(AMPS-*co*-AAm) is best described by the Langmuir model, consistent with monolayer chemisorption driven by electrostatic interactions between the cationic dye and the anionic sulfonate groups of the hydrogel.

#### 3.2.4. Adsorption kinetics

To elucidate the rate-controlling mechanism of MG adsorption onto the p(AMPS-*co*-AAm) hydrogel, the experimental kinetic data were fitted using PFO and PSO models.^37^ The linearized forms are given by eqn (10) and (11), respectively, as follows:

10 ln(*q*_e_ − *q*_t_) = ln *q*_e_ − *K*_1_*t*,

11
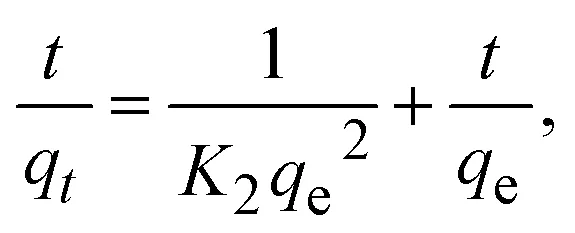
where *K*_1_ (min^−1^) is the PFO rate constant, *K*_2_ (g mg^−1^ min^−1^) is the PSO rate constant, *q*_t_ (mg g^−1^) is the adsorption capacity at time *t* (min), and *q*_e_ (mg g^−1^) is the equilibrium adsorption capacity.

**Fig. 7:**
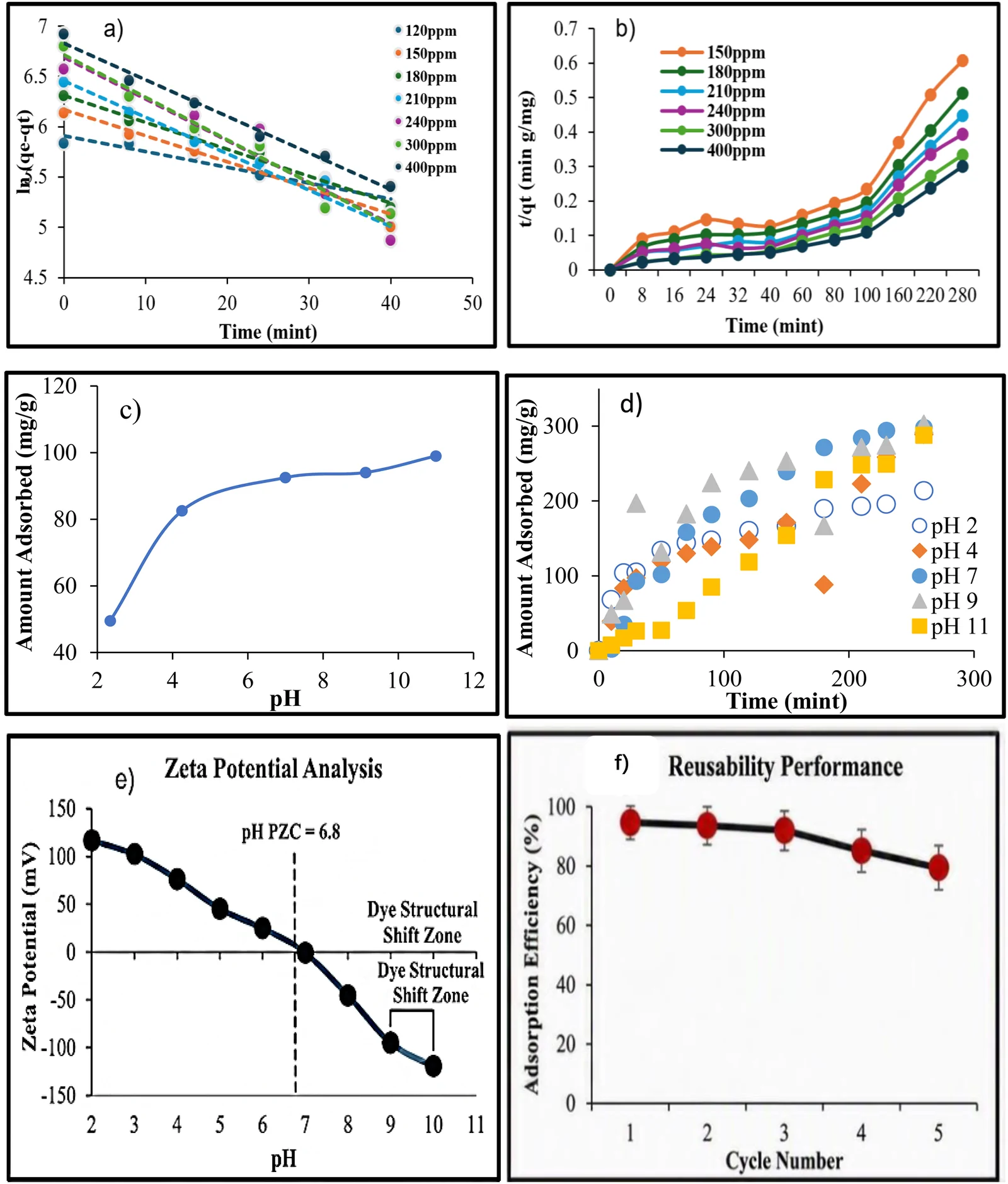
(a) PFO kinetic plot of ln *q*_e_ − *q*_t_*versus* time (min). (b) PSO kinetic plot of *t*/*q*_t_*versus* time (min). (c) Effect of the solution pH on the MG adsorption capacity of the p(AMPS-*co*-AAm) hydrogel. (d) Adsorption capacity *versus* time at different pH values. Experimental conditions: 0.03 g p(AMPS-*co*-AAm), 100 mL MG solution (120 ppm), pH = 2, 4, 7, 9, and 11, and 25 °C. (e) Zeta potential analysis of the p(AMPS-*co*-AAm) hydrogel and malachite green dye. (f) Reusability of the adsorbent for five cycles using a 0.1 M HCl solution as the desorption medium.

The PFO model was evaluated from a linear plot of ln (*q*_e_ − *q*_t_) *versus t* (Fig. 7a), and the PSO model from a plot of *t*/*q*_t_*versus t* (Fig. 7b). Table S3 and S4 represent the parameters of PFO and PSO kinetics models, respectively. This data showed that the *R*^2^ values obtained from PFO models were closer to unity as compared to those obtained from the PSO models. Similarly, the values of *q*_e_ calculated from the PFO models ware closer to experimental values, while those obtained from the PSO models were far away from the experimental data. These findings suggested that MG adsorption onto p(AMPS-*co*-AAm) followed the pseudo-first-order kinetics.

The overall process is therefore physisorption-controlled in terms of kinetics, while the Langmuir isotherm analysis (Section 3.2.3) independently confirms monolayer surface coverage consistent with a chemisorptive equilibrium state; both findings being mutually compatible and well-precedented in the literature on hydrogel adsorption.

#### 3.2.5. Impact of pH on MG adsorption

The impact of the solution pH on MG removal was investigated across pH values of 2, 4, 7, 9, and 11, with a fixed hydrogel dosage of 0.03 g and an initial MG concentration of 120 ppm at 25 °C. As shown in Fig. 7c and d, *q* increased with the increasing pH, reaching a maximum of 302.33 mg g^−1^ and a removal efficiency of 98.99% at pH 11. This behavior is consistent with the anionic character of the p(AMPS-*co*-AAm) hydrogel: at low pH, the protonation of the sulfonate and amide groups partially suppresses the negative surface charge, reducing electrostatic attraction toward the cationic MG molecule. At alkaline pH, deprotonation enhances the anionic character of the hydrogel surface, strengthening electrostatic interactions and thereby improving dye uptake. These findings confirm that the solution pH is a critical operational parameter governing the MG adsorption efficiency under optimal alkaline conditions for this system. Fig. 7e represents the zeta potential of the p(AMPS-*co*-AAm) hydrogel as a function of pH. The hydrogel exhibits a clear transition from positive values at low pH to significantly negative values at high pH, with the point of zero charge (pH_PZC_) identified around pH 6.8. This confirms that the deprotonation of p(AMPS-*co*-AAm) with the increase in pH actually enhances electrostatic attraction with the cationic MG molecules during the adsorption process. The high apparent adsorption under highly alkaline conditions of pH 11 may partially be influenced by the structural fading and chemical transformation of the MG dye into its neutral carbinol form, rather than being driven entirely by adsorption.

#### 3.2.6. Reusability of adsorbents

The regeneration and reuse study of the p(AMPS-*co*-AAm) adsorbent was performed by using 0.1 M HCl solution as a desorption medium. The adsorbent was regenerated by desorption of the adsorbed dye followed by washing with distilled water. The regenerated adsorbent was reused to adsorb MG under the same conditions. This process of regeneration and reuse was performed five times in a row, and the corresponding results are shown in Fig. 7f. A 20% decrease in the adsorption efficiency was observed upon reusing the adsorbent for the fifth cycle. This decrease in efficiency may be primarily associated with the attachment of impurities (present in dyes) on the adsorption active sites of the hydrogel. In addition, the permanent entrapment of few dye molecules in the hydrogel network may not be neglected to contribute to the decrease in efficiency upon regeneration.

### 3.3. Mechanism of adsorption

**Fig. 8:**
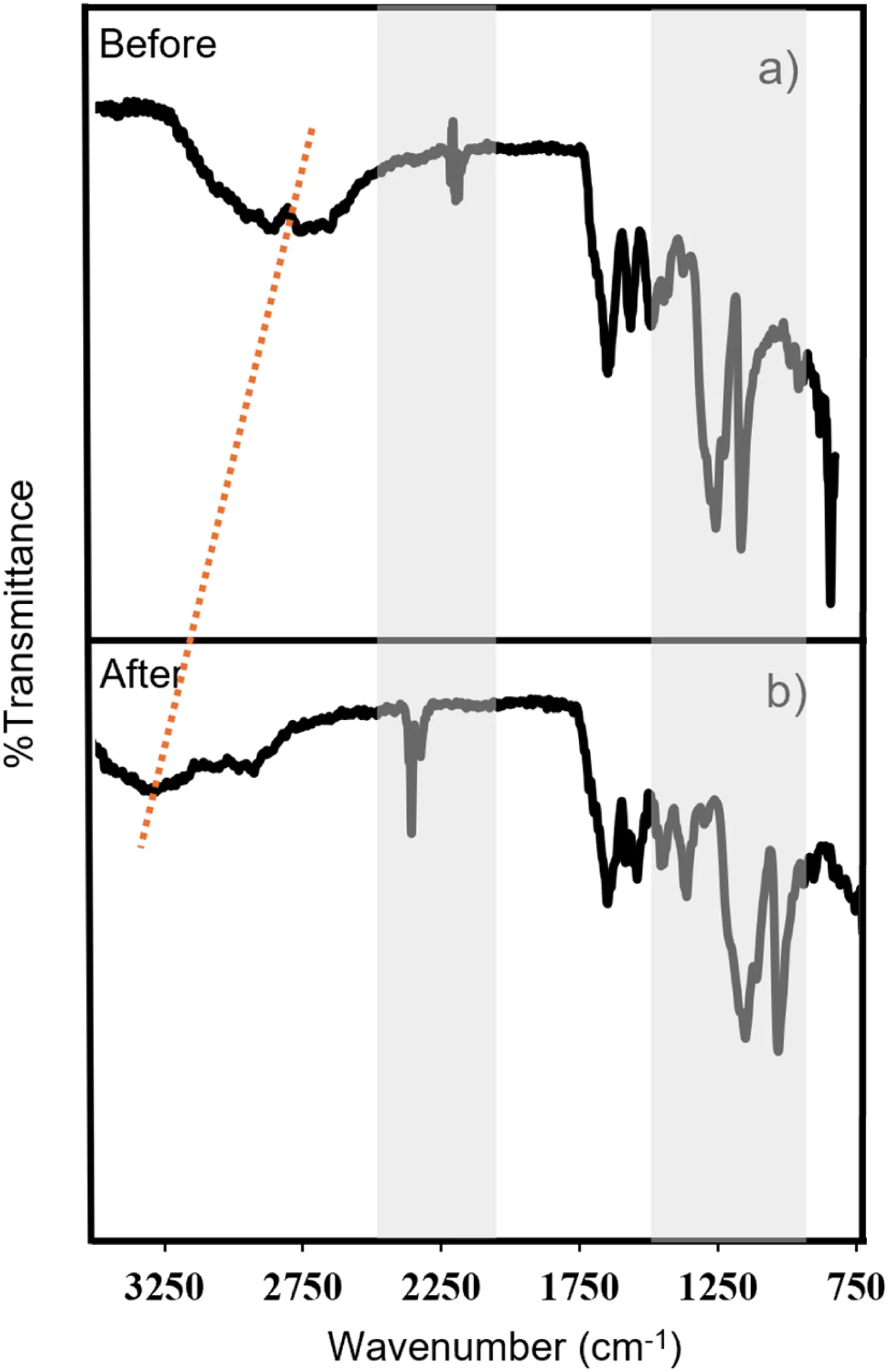
(a) FTIR spectrum of p(AMPS-*co*-AAm). (b) FTIR spectrum showing the structural changes in the p(AMPS-*co*-AAm) hydrogel after malachite green dye adsorption by the hydrogel.

To understand the mechanism of adsorption of MG on the p(AMPS-*co*-AAm) hydrogel, FTIR analysis of hydrogel was carried out before and after adsorption, and the corresponding spectra are shown in Fig. 8. In the FTIR spectra of the pristine hydrogel, a broad peak in the region of 3000–3500 cm^−1^ may be attributed to the overlapping effect from the stretching vibrations of N–H- and H-bonded water molecules in the polymer matrix. The characteristic bands of sulfonate S=O symmetric and asymmetric stretches appear in the region of 1200–1440 cm^−1^.

**Fig. 9:**
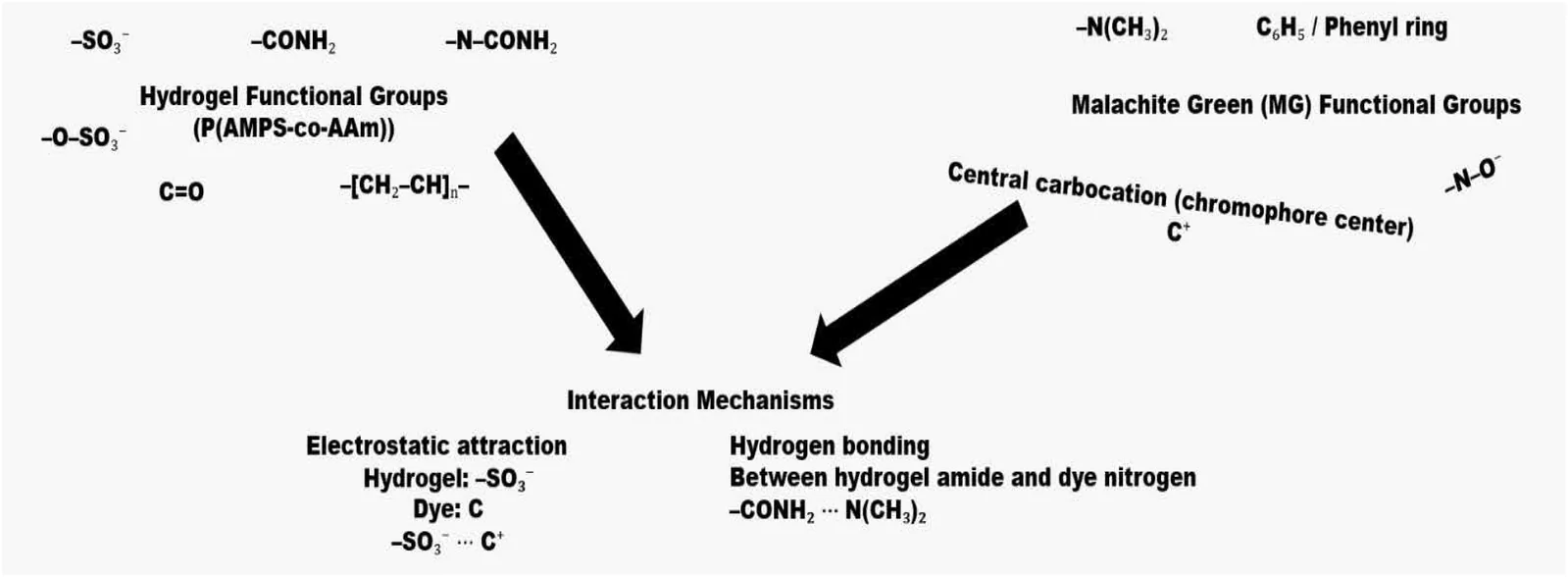
Schematic of the mechanism of dye adsorption.

After adsorption of the dye, a noticeable shift in the S=O peak was observed that may be attributed to the electrostatic interactions between the anionic sulfonate group and the cationic MG dye. Moreover, new absorption features emerging in the 1600–1400 cm^−1^ region are ascribed to the aromatic C=C stretching and C–N vibrations of the triphenylmethane chromophore of MG, confirming successful dye adsorption. The retention of all the principal backbone peaks confirms the retention of the structural integrity of the hydrogel. Overall, the spectral analysis indicates that the main mechanism of adsorption is the strong attraction between the positively charged carbocation center of MG and the negatively charged sulfonate groups (SO_3_^−^) of the hydrogel's network. In addition, the hydrogel's amide groups (–CONH_2_ and –CONH) improve the adsorption stability by forming hydrogen bonds with the dye's dimethylamino groups. The schematic of the adsorption of malachite green dye onto the p(AMPS-*co*-AAm) hydrogel is given in Fig. 9.

### 3.4. Comparison with the literature

Table 1 represents the comparison of the adsorption capacity of the p(AMPS-*co*-AAm) hydrogel synthesized in this work with recently reported hydrogel adsorbents for the removal of MG from water. Researchers have used hydrogels of different compositions such as those containing alginate, lignin, starch, galactomannan gum, and their composites graphene oxide (GO) and manganese oxide (MnO_2_) for the adsorptive removal of MG from water. The comparison shows that the adsorption capacity of the p(AMPS-*co*-AAm) hydrogel reported in this work is far better than these previously reported hydrogel adsorbents. This comparatively high adsorption capacity may be achieved by the synergistic effects of the functional groups (sulfonic and amide) present in this hydrogel and its light crosslinking, which allows the swelling of the hydrogel network to capture a higher amount of MG.

**Table 1:** Comparison of the adsorption capacity of the p(AMPS-*co*-AAm) hydrogel with other materials reported in the recent literature

Sr. No.	Adsorbent	Adsorption capacity (mg g^−1^)	Reference
1	Graphite modified sodium alginate hydrogel	628.93	38
2	Sodium alginate/silica-ZnO	322.58	39
3	Lignin- and black liquor-based composite hydrogels	650	40
4	Fenugreek galactomannan gum MnO_2_ hydrogel	526.31	41
5	Hydroxyethyl starch-graphene oxide hydrogel	89.3	42
6	Poly(AMPS-*co*-AAm) hydrogel	930.31	This work

## 4. Conclusions

This study reveals the use of the bulk p(AMPS-*co*-AAm) hydrogel as an effective adsorbent for the removal of malachite green dye from aqueous solutions. The hydrogel was successfully synthesized *via* free radical polymerization and exhibited a highly porous, interconnected morphology, as confirmed by SEM analysis, alongside an exceptionally high equilibrium swelling capacity of 2009.49% and a water content of 95.25%, both of which are favorable for adsorption applications in aqueous media. Water transport through the hydrogel network was found to follow a non-Fickian mechanism, with swelling kinetics best described by the pseudo-first-order model. Adsorption was examined under different conditions of adsorbent amount, initial dye concentration, contact duration, and pH level. Under the optimized conditions, a peak adsorption capacity of 930.31 mg g^−1^ was attained at 400 ppm of MG, resulting in nearly total removal (99.30%) of the dye. Isotherm analysis verified that the MG dye adheres to the Langmuir model, suggesting monolayer adsorption on a smooth surface with consistent binding energy, aligning with the electrostatic interactions between the negatively charged hydrogel surface and the positively charged MG dye. Additionally, kinetic modelling indicated PFO as the most suitable option, implying that the rate largely relies on the accessibility of active sites. Together, these results position bulk p(AMPS-*co*-AAm) as a potential, effective and cost-efficient adsorbent for eliminating cationic pollutants from wastewater.

## Conflicts of interest

The authors declare that they have no known competing financial interests or personal relationships that could have appeared to influence the work reported in this paper.

## Supplementary Material

RA-016-D6RA05581H-s001

## Data Availability

The authors declare that the data are available in this manuscript and its supplementary information (SI) file in the form of tables and figures. Supplementary information is available. See DOI: https://doi.org/10.1039/d6ra05581h.
