# Supplementary material for: Adsorptive removal of the cationic dye malachite green from wastewater with an anionic hydrogel
Source: RSC Adv. 2026 Aug 24;16(41):49446–58. doi: 10.1039/d6ra05581h (PMC13501377; doi:10.1039/d6ra05581h)
Supplement: Supplementary file 1 [file RA-016-D6RA05581H-s001.pdf]

Supplementary Information (SI) for RSC Advances.
This journal is © The Royal Society of Chemistry 2026

Adsorptive removal of cationic dye malachite green from wastewater with anionic hydrogel

Sawera Safdar[1], Muhammad Ajmal[1], Muhammad Abid Zia[1*], Kashif Ali Khan[2], Afzal Shah[3*]

[1]Department of Chemistry, University of Education Lahore, Attock Campus, Punjab, 43600, Pakistan

[2]Department of Chemical and Environmental Engineering, University of Nottingham, Ningbo 315100, China

[3]Department of Chemistry, Quaid-i-Azam University, Islamabad, 45320, Pakistan

*Correspondence: afzals_qau@yahoo.com (A. Shah)

abid@ue.edu.pk (M.A. Zia)

S1. Chemical reagents/ Chemicals employed:

Acrylamide (AAm) served as the monomer and N,N'-methylenebisacrylamide (MBA) as the crosslinking agent in the formation of "Poly(AMPS-co-AAm) gel matrix" that was carried out for the whole experiments. The polymerization was started with ammonium persulfate (APS), and the reaction was accelerated with N,N,N',N'-tetramethylethylenediamine (TEMED). The model dye for the adsorption investigations was malachite green (MG). Throughout, distilled water served as both the cleaning agent and the reaction medium.

Table S1. Following is the summary of materials used including the formulas and molecular mass.

Sr. No	Chemicals	Structure	Molar weight (g/mol)
1	Acrylic acid (AAc)	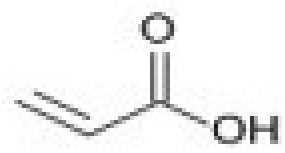	72.06
2	Sodium borohydride ($NaBH_4$)	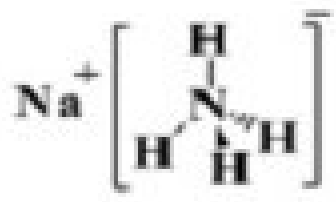	37.83
3	N,N'-methylenebisacrylamide(MBA)	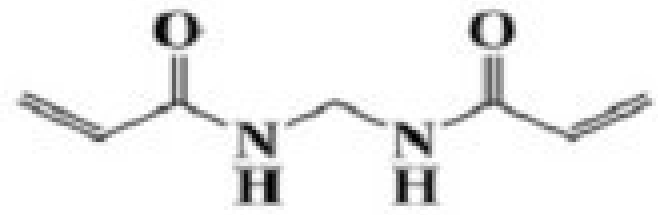	154.17
4	Ammonium persulphat(APS)	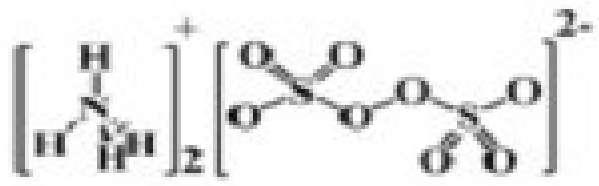	228.21
5	N,N,N',N'-tetramethylethylene 1,2-diamine (TEMED)	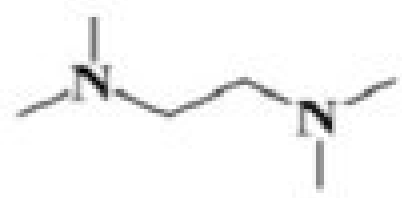	116.208

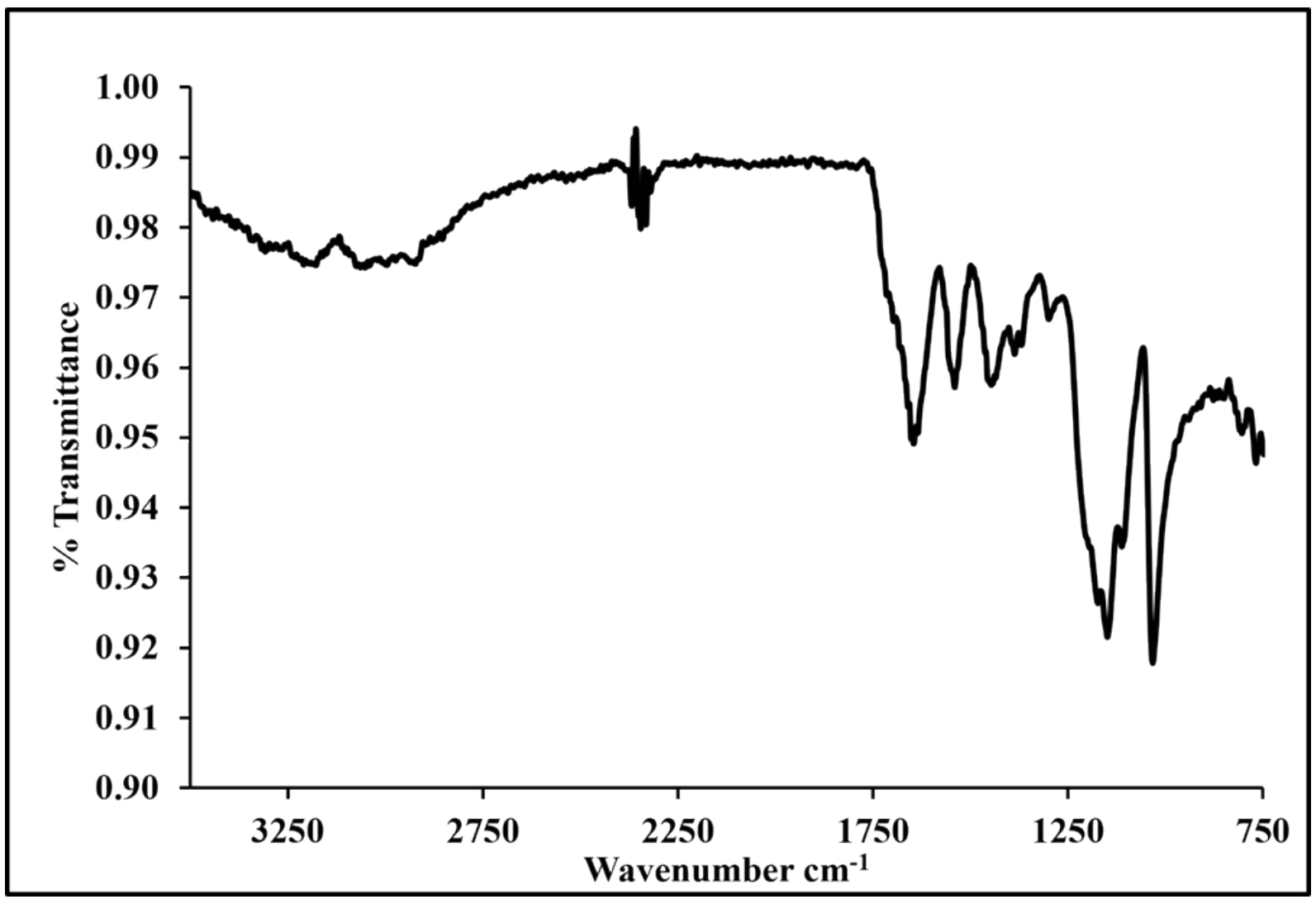

Figure S1. FTIR spectral analysis of p(AMPS-co-AAm)**.**

Table S2. Langmuir, Freundlich, and Temkin isotherm parameters for MG adsorption onto p(AMPS-co-AAm) hydrogel at 25 °C.

Langmuir Isotherm Constants		
K_L (L/g)	q_m (mg/g)	R^2
0.065693	1111.111	0.9918
**Freundlich Isotherm Constants**		
K_F (L/g)	N	R^2
174.5131	2.499375	0.8738
**Temkin Isotherm Constants**		
K_T (L/g)	B	R^2
0.580199	248.96	0.9259

Table S3. The values of kinetic parameters derived from PFO kinetics model.

Initial Concentration		Constants		Experimental
**MG dye**	**K_1(min^{-1})**	**q_e(mg/g)**	**R^2**	**q_e(mg/g)**
**120 ppm**	0.0156	368.153	0.923	340.87
**150 ppm**	0.026	478.377	0.943	461.239
**180 ppm**	0.0268	550.375	0.989	546.318
**210 ppm**	0.0363	635.302	0.966	625.989
**240 ppm**	0.0415	803.358	0.937	711.984
**300ppm**	0.0423	822.296	0.967	839.990
**400ppm**	0.0363	921.774	0.987	930.307

Table S4. The kinetic parameters derived from the Pseudo Second-Order (PSO) model for the adsorption of malachite green dye onto the bulk P(AMPS-co-AAm)

hydrogel.

Initial Conc.	Constants			Experimental
**MG dye**	**k_2 ($g.mg^{-1}.min^{1}$)**	**q_e (mg/g)**	**R^2**	**q_e (mg/g)**
**120 ppm**	0.087	18.832	0.702	340.87
**150 ppm**	0.029	22.123	0.813	461.2393
**180 ppm**	0.022	26.385	0.825	546.318
**210 ppm**	0.016	28.901	0.83	625.989
**240 ppm**	0.016	32.467	0.823	711.984
**300 ppm**	0.011	36.63	0.856	839.9902
**400 ppm**	0.009	42.373	0.829	930.307

S2. Instruments

UV-Vis spectrophotometer (**YOKE N6000,** Lambda-25), a weighing balance (**0.001g** and maximum capacity of **220 g** was used for accurate measurement of chemicals and hydrogel samples) were used throughout the experimental procedures.

S3. Swelling capacity Assessment

Three dried gel samples were weighed and measured individually and then immersed in 150 mL of water. Mass was recorded after every 30 minutes until equilibrium was reached. Swelling curve was plotted against time interval.
